# The potential risks of equine serum therapy in transmitting new infectious diseases: lessons from a post-pandemic era

**DOI:** 10.3389/fpubh.2024.1366929

**Published:** 2024-02-14

**Authors:** Manuela B. Pucca, Ana Lucia Camphora

**Affiliations:** ^1^Department of Clinical Analysis, School of Pharmaceutical Sciences, São Paulo State University (UNESP), Campus Araraquara, São Paulo, Brazil; ^2^Independent Researcher on Intersections of Modern and Contemporary Equine Cultures, Rio de Janeiro, Brazil

**Keywords:** infectious diseases, viral transmission, pandemic risks, infection control measures, health crisis, heterologous serum therapy

In the world of medical treatments, certain interventions carry hidden risks that are not always readily apparent. The use of equine serum in human therapies has raised substantial concerns, often overlooking, or minimizing the potential risks associated with these interventions. One prime example is the use of equine-derived antivenoms, crucial in treating venomous animal envenomations, such as those caused by snakes, scorpions, and spiders. A primary concern centers around the immunogenicity of equine serum components upon introduction into the human body. This can provoke immune responses ranging from mild allergic reactions to serum sickness and severe anaphylaxis, necessitating immediate medical intervention (1).

Likewise, equine serum-derived treatments may carry the risk of transmitting infections or diseases from the horse to the human recipient. The purification process involves pasteurizing horse IgGs. Typically, pasteurization occurs in the presence of stabilizers such as amino acids, sugars, or citrate to preserve protein functionality, preventing molecular changes and protein aggregation. These stabilizers also contribute to fortifying against viruses, underscoring the need to validate treatment conditions. Pasteurization can effectively deactivate a variety of viruses, both enveloped and non-enveloped (e.g., HIV, HBV, HCV, and HAV). However, there is limited data on the inactivation of resistant non-enveloped viruses like porcine parvovirus, SV 40, or reovirus type 3 in plasma products (2, 3). Thus, pasteurization has proven itself the only effective step toward assuring the virus safety of final product (4), although it is a process that do not have deliberately introduced viral inactivation, that could result in the parenteral transmission of zoonotic diseases. Caprylic acid treatment, formulation at acidic pH, and ion-exchange chromatography represent additional purification steps employed in the process. While these methods can effectively remove viruses from the serum, it's worth noting that they may not be as robust or comprehensive in their virus-removal capabilities (3, 5, 6).

Recent occurrences of zoonotic diseases serve as indicators of the interplay between humans and the reservoirs of biological agents harbored by animals (7). Moreover, these events underscore the inherent perils associated with the emergence of novel diseases such as the human immunodeficiency virus (HIV), Hantaan, Lassa, Ebola, Nipah, and a variety of paramyxoviruses. Furthermore, the list expands to encompass the equine morbilli virus, the West Nile virus, and notably, the strong likelihood of the inclusion of the severe acute respiratory syndrome coronavirus, including SARS-CoV-2 (8, 9). It is also important to highlight the actual endemic status of hepatitis E (HEV), which represents a significant public health concern. The transmission dynamics of this disease have been found to extend beyond conventional routes, potentially including blood transfusions as an important mode of spread (10).

Burnouf et al. (3) have put forward a list of 19 viruses, with the capacity to cause diseases in horses, and notably, 11 of these can also induce diseases in humans. The shared characteristics, encompassing attributes such as being enveloped or non-enveloped, DNA or RNA-based, etc., are depicted in Figure 1. Notably, Bornouf has proposed to include screening for at least these specific pathogens for sera derived from horses; however, these recommendations have not been implemented to date (3).

**Figure 1:**
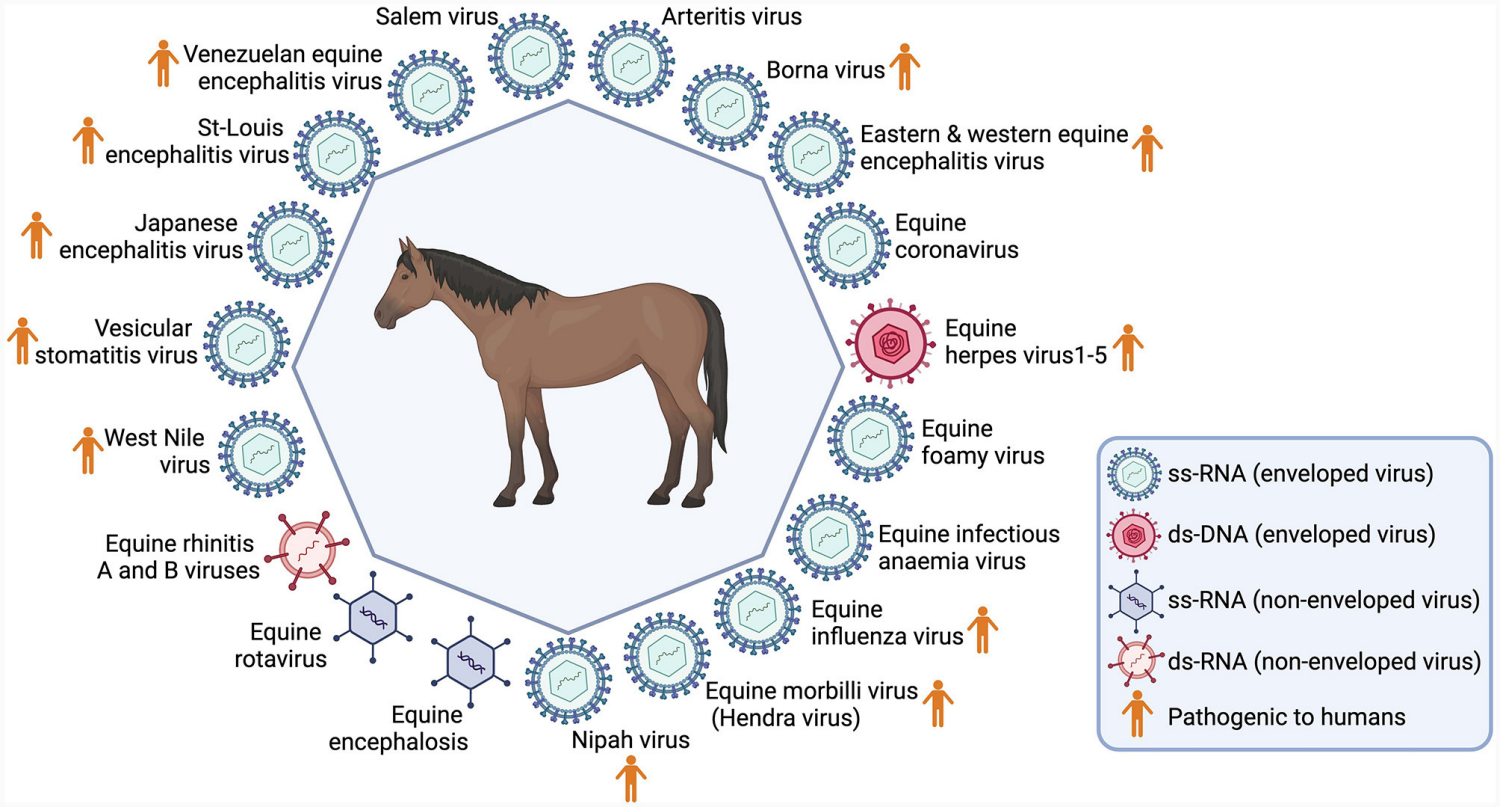
Main viruses identified in horses. Created with Biorender.com.

In fact, diseases like Eastern equine encephalitis (EEE), Western equine encephalitis (WEE), and Venezuelan-equine encephalitis (VEE) are highly infectious, spreading through aerosols. Venezuela and Colombia have seen continuous, fatal cases of VEE-induced encephalitis in horses and humans (11). On the other hand, Vesicular Stomatitis (VS), common among North American horses, poses zoonotic risks by causing encephalitis in children (12); while Hendra Virus (HeV) leads to respiratory and neurological diseases, fatal for humans and horses (13). West Nile fever has recently spread across new territories globally (14), being considered a epizootic emergence (15). Although rare in horses, rabies remains a grave public health concern (16, 17), in contrast with Equine Influenza, which apparently do not affect humans (18). Regarding SARS-CoV-2, although there is little evidence for horse natural infection (19), it was evidenced that a COVID-19 patient infected a horse, demonstrating in the horse the seroconversion following dayle contact during the development of clinical disease (20).

However, the diseases mentioned earlier are only the ones we're aware of; there could be numerous others being transmitted. In fact, it is estimated that 60% of emerging infectious diseases that are reported globally are zoonoses and over 30 new human pathogens have been detected in the last three decades, 75% of which have originated in animals (21).

This situation brings up a puzzling question: If we're not sure about the different diseases that could be in equine-derived serum, how can we know what illnesses might spread from it? Screening equine-derived serum for potential diseases with zoonotic implications may requires a multifaceted approach encompassing various techniques, including virus-specific PCR assays, serological tests, metagenomic sequencing, mass spectrometry, viral culture, next-generation sequencing (NGS), microarray analysis, immunofluorescence assays (IFA), proteomic analysis, nucleic acid amplification techniques (NAAT), cell culture-based assays, bioinformatics and computational analysis, lateral flow assays, digital PCR, and/or biosensors. As they are not applied so far, using serum from horses might not just bring new diseases to human but also create big outbreaks like the recent pandemic of COVID-19. This careless use of animal-based serums, such horses, is like “playing a risky game with public health”. Not being careful with these treatments ignores lessons from history and science.

We must not overlook the absence of transparency concerning these risks for patients and their families. Patients have the right to informed decision-making about their treatment choices, empowered by a thorough grasp of potential advantages and risks. And in this context, no information beyond the most common and rare side effects (i.e.,; allergies, serum sickness, fever, and anaphylaxis), that include the risks of contracting diseases, is described in the antivenom's label (22).

In the midst of the 21st century, with unprecedented achievements in science and pharmaceuticals, and with the glaring lessons imparted by the COVID-19 pandemic, one might wonder how we continue to endorse the archaic practice of using equine serum as a therapeutic option.

In the pursuit of alternatives to serum derived from horses, notable progress has been achieved, with a particularly promising pathway being the utilization of human monoclonal antibodies generated through phage display technology (23). The production of human monoclonal antibodies via phage display offers distinct advantages, including diminished immunogenicity and the capacity to customize antibodies for therapeutic applications (1). This methodology not only reduces dependence on equine serum but also provides a more individualized and human-centric solution, underscoring the transformative potential of cutting-edge technologies in advancing biotechnological alternatives (24). Currently, numerous researchers are actively exploring monoclonal antibodies and other alternatives to replace serum derived from horses (25–32).

While medical breakthroughs surge ahead, it is disconcerting that we overlook the pressing need to rigorously scrutinize the safety of treatments, particularly those produced by animal sources. Ignoring this matter not only dismisses the significant advancements in medical understanding but also puts the fundamental aspects of human health and wellbeing in danger.

## Author contributions

MP: Writing—original draft, Writing—review & editing. AC: Writing—original draft, Writing—review & editing.
